# Supplementary figures and images for: Statistical methods to disentangle genetic effects influencing infertility and early fetal viability with a genome-wide application
Source: PLoS Genet. 2025 Dec 1;21(12):e1011952. doi: 10.1371/journal.pgen.1011952 (PMC12680335; doi:10.1371/journal.pgen.1011952)

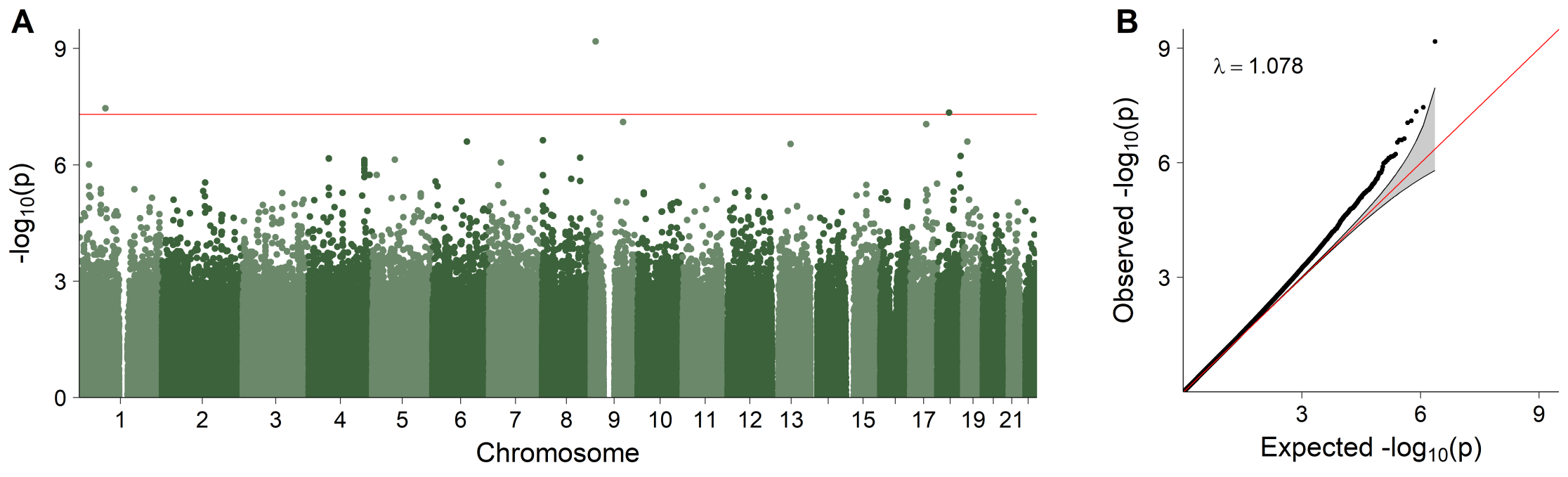

Supplement: S1 Fig — Panel A displays the negative log10-transformed p-values, and the red line indicates the Bonferroni-corrected genome-wide significance threshold of 5 × 10^−8^. Panel B shows the corresponding quantile-quantile (QQ) plot, along with the genomic inflation factor (λ). (TIF) [file pgen.1011952.s004.tif]

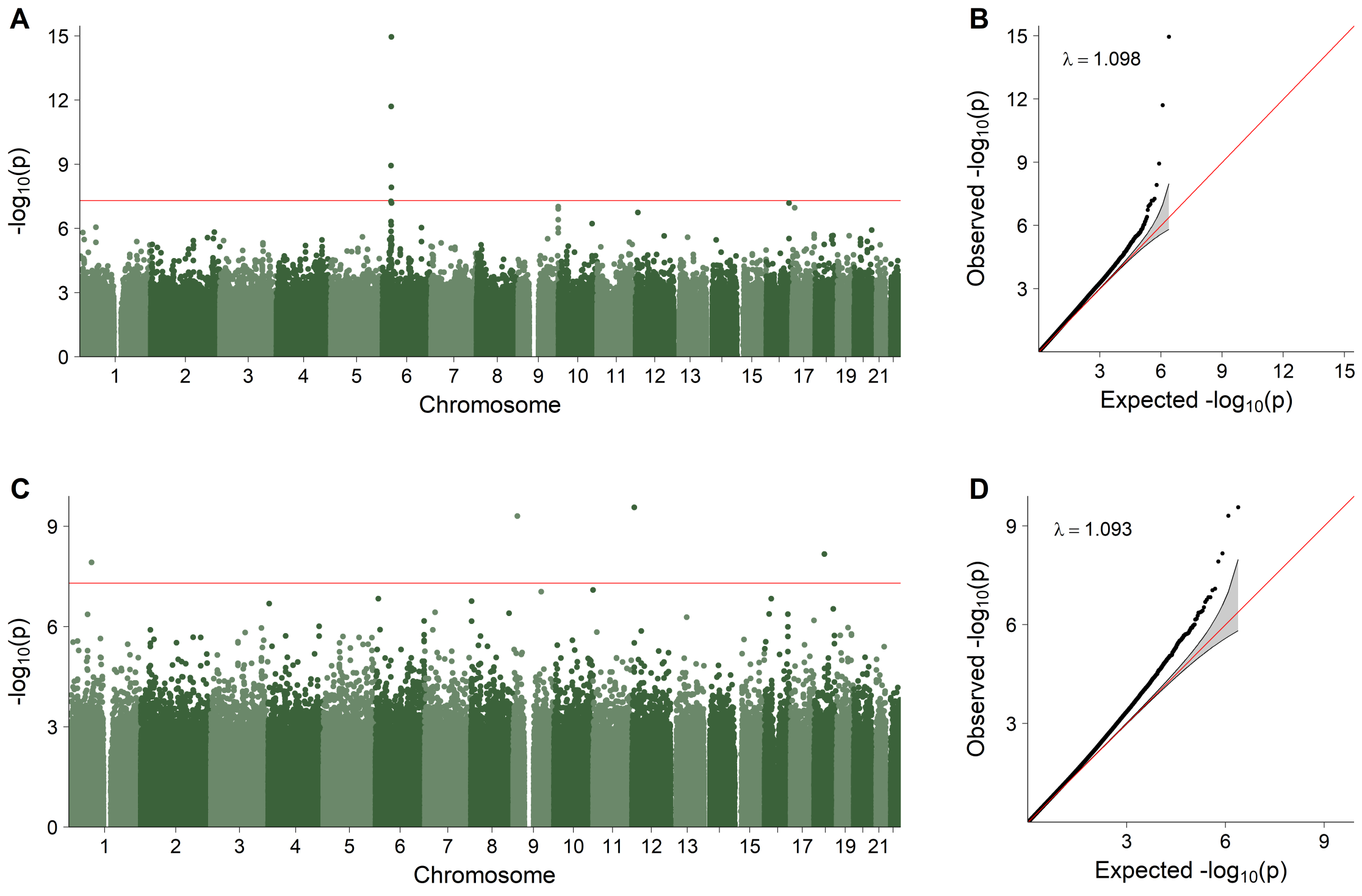

Supplement: S2 Fig — Panels A and C display the negative log10-transformed p-values, and the red line indicates the Bonferroni-corrected genome-wide significance threshold of 5×10^−8^. Panels B and D show the corresponding quantile-quantile (QQ) plots, along with the genomic inflation factor (λ). (TIF) [file pgen.1011952.s005.tif]

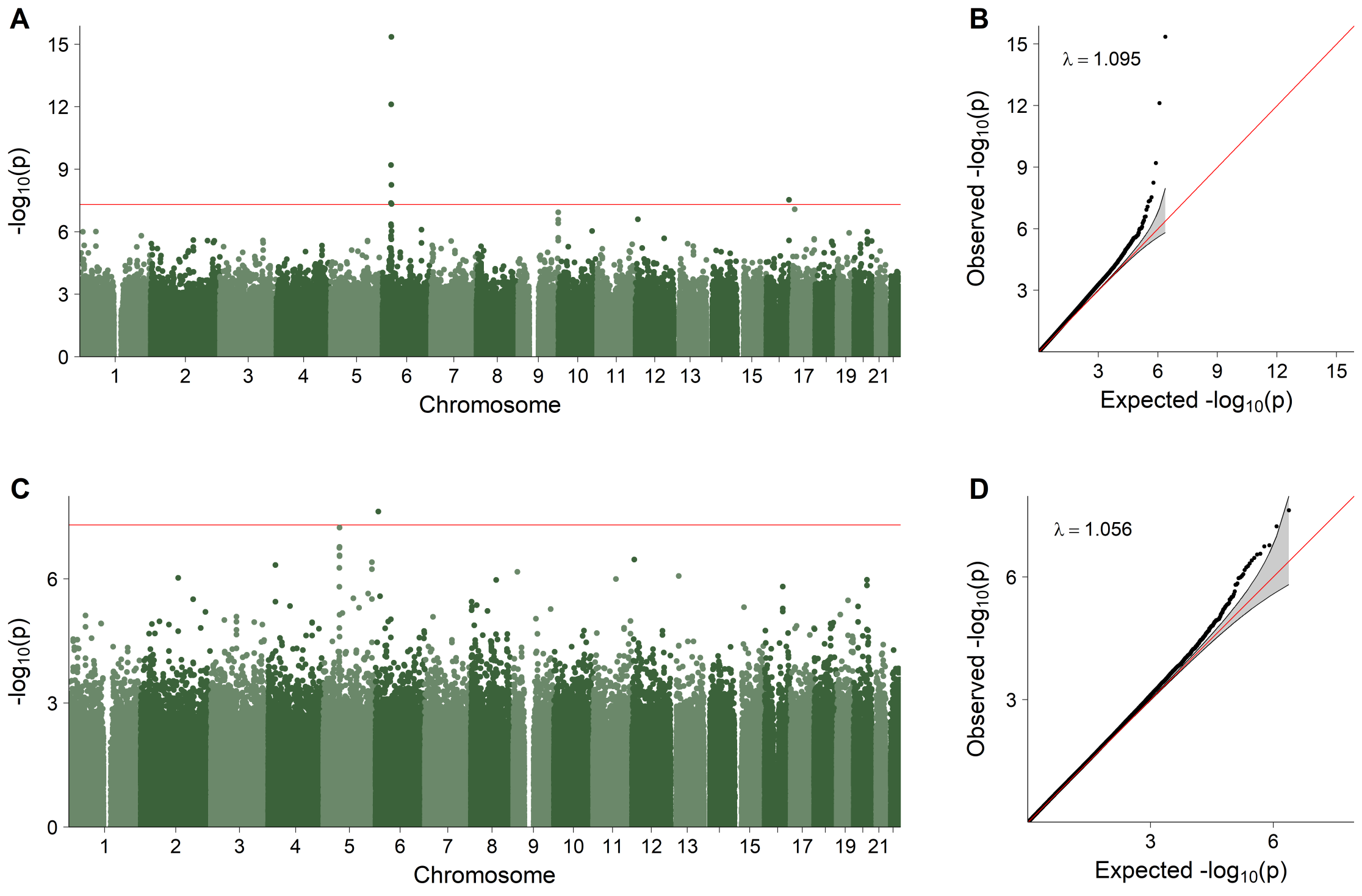

Supplement: S3 Fig — Panels A and C display the negative log10-transformed p-values, and the red line indicates the Bonferroni-corrected genome-wide significance threshold of 5×10^−8^. Panels B and D show the corresponding quantile-quantile (QQ) plots, along with the genomic inflation factor (λ). (TIF) [file pgen.1011952.s006.tif]

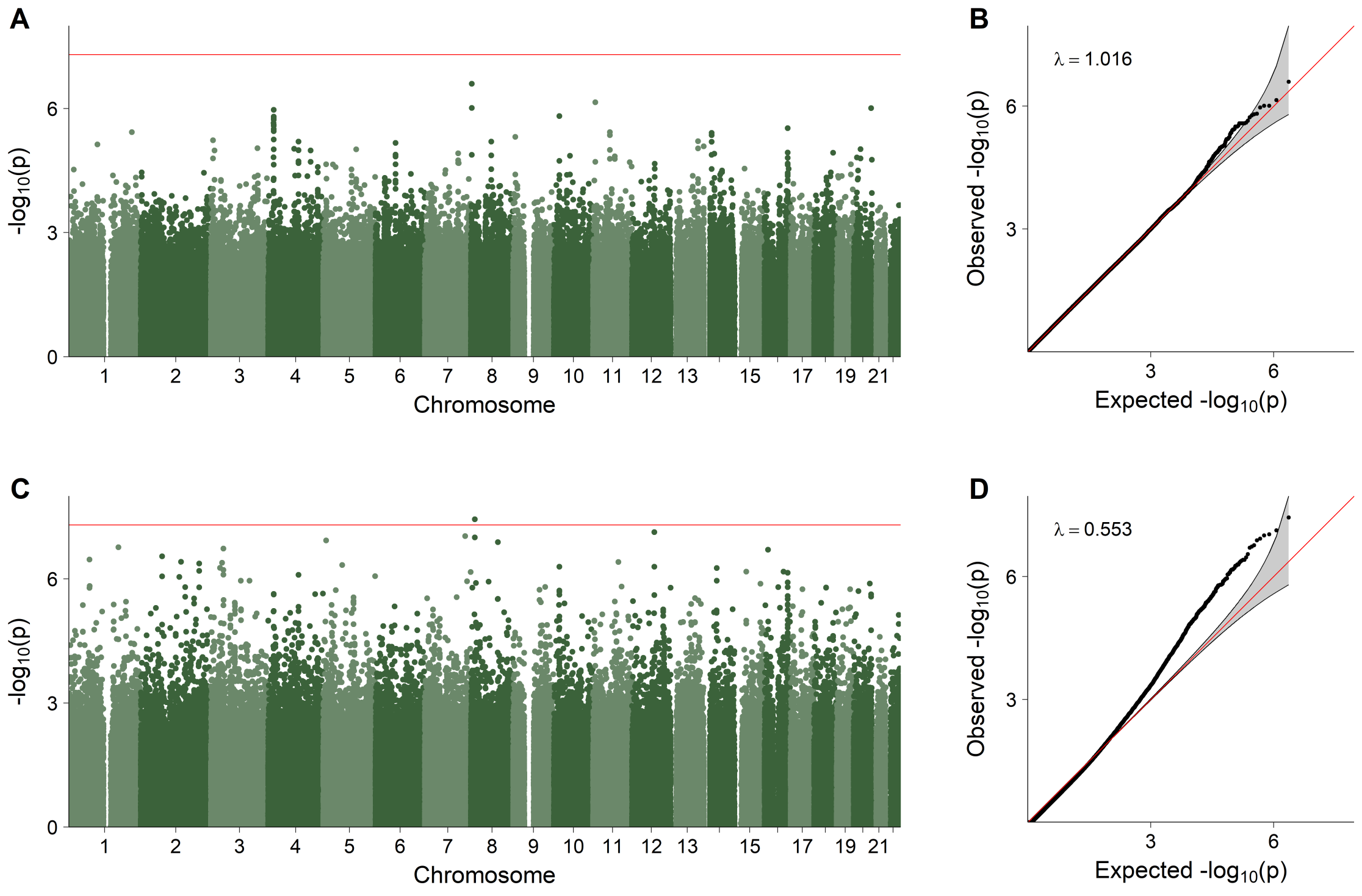

Supplement: S4 Fig — Panels A and C display the negative log10-transformed p-values, and the red line indicates the Bonferroni-corrected genome-wide significance threshold of 5×10^−8^. Panels B and D show the corresponding quantile-quantile (QQ) plots, along with the genomic inflation factor (λ). (TIF) [file pgen.1011952.s007.tif]

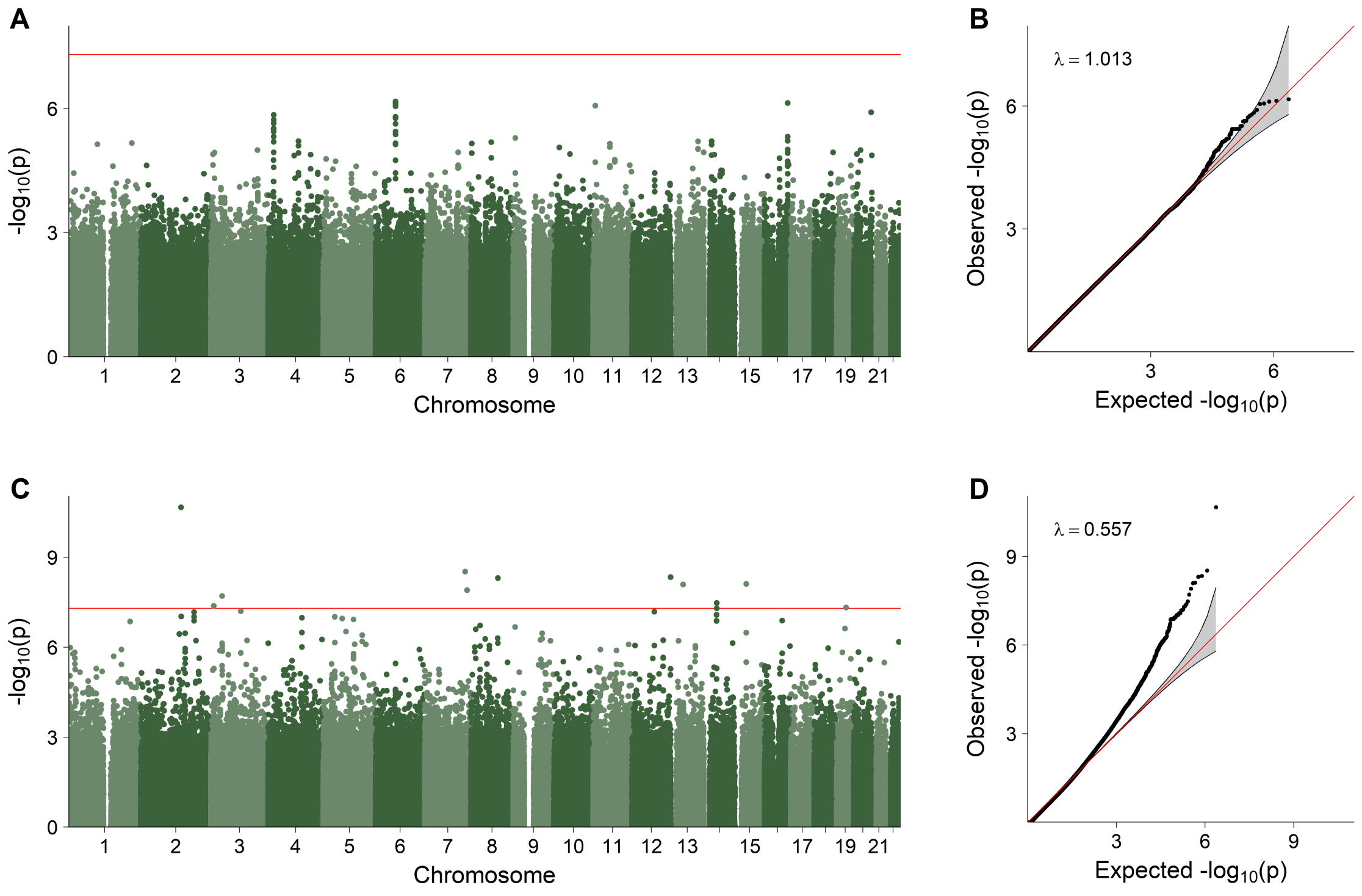

Supplement: S5 Fig — Panels A and C display the negative log10-transformed p-values, and the red line indicates the Bonferroni-corrected genome-wide significance threshold of 5×10^−8^. Panels B and D show the corresponding quantile-quantile (QQ) plots, along with the genomic inflation factor (λ). (TIF) [file pgen.1011952.s008.tif]

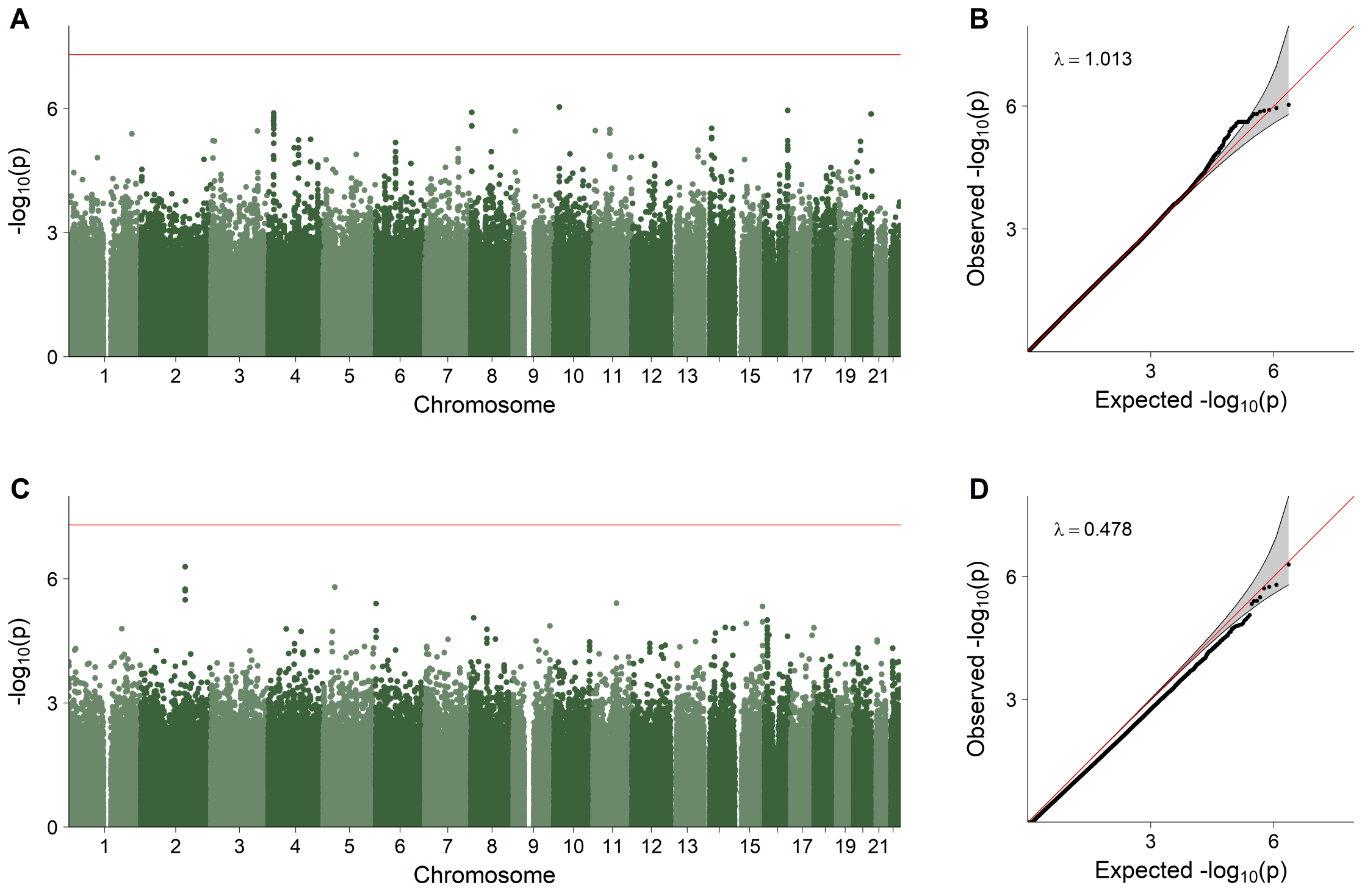

Supplement: S6 Fig — Panels A and C display the negative log10-transformed p-values, and the red line indicates the Bonferroni-corrected genome-wide significance threshold of 5×10^−8^. Panels B and D show the corresponding quantile-quantile (QQ) plots, along with the genomic inflation factor (λ). (TIF) [file pgen.1011952.s009.tif]

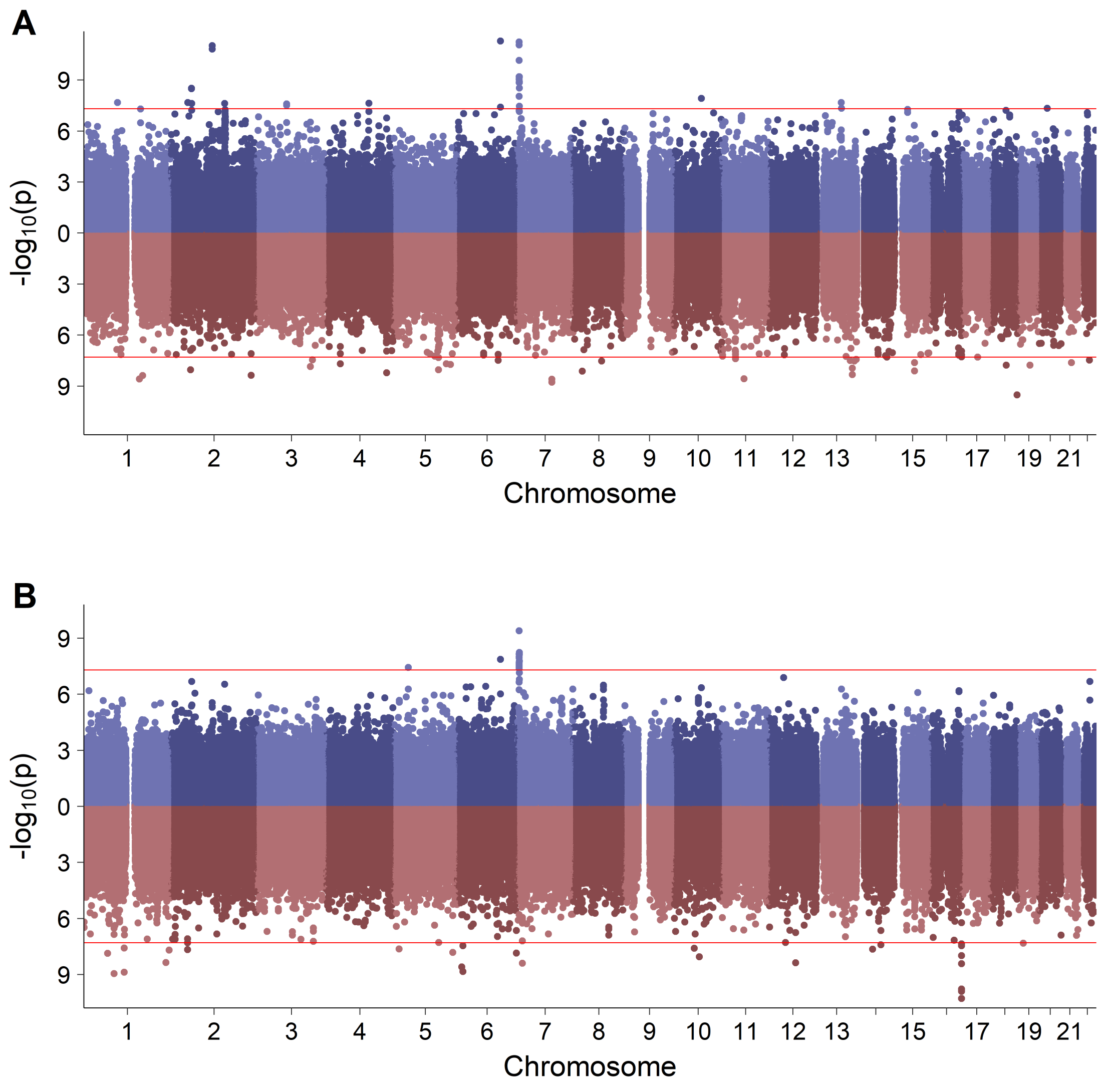

Supplement: S8 Fig — The parental interaction effect are shown for the complementary model (panel A) and the threshold-based model (panel B) in the ART sample (blue) and the non-ART sample (red). The negative log10-transformed p-values are displayed, and the red lines indicate the Bonferroni-corrected genome-wide significance threshold of 5×10^−8^. (TIF) [file pgen.1011952.s011.tif]

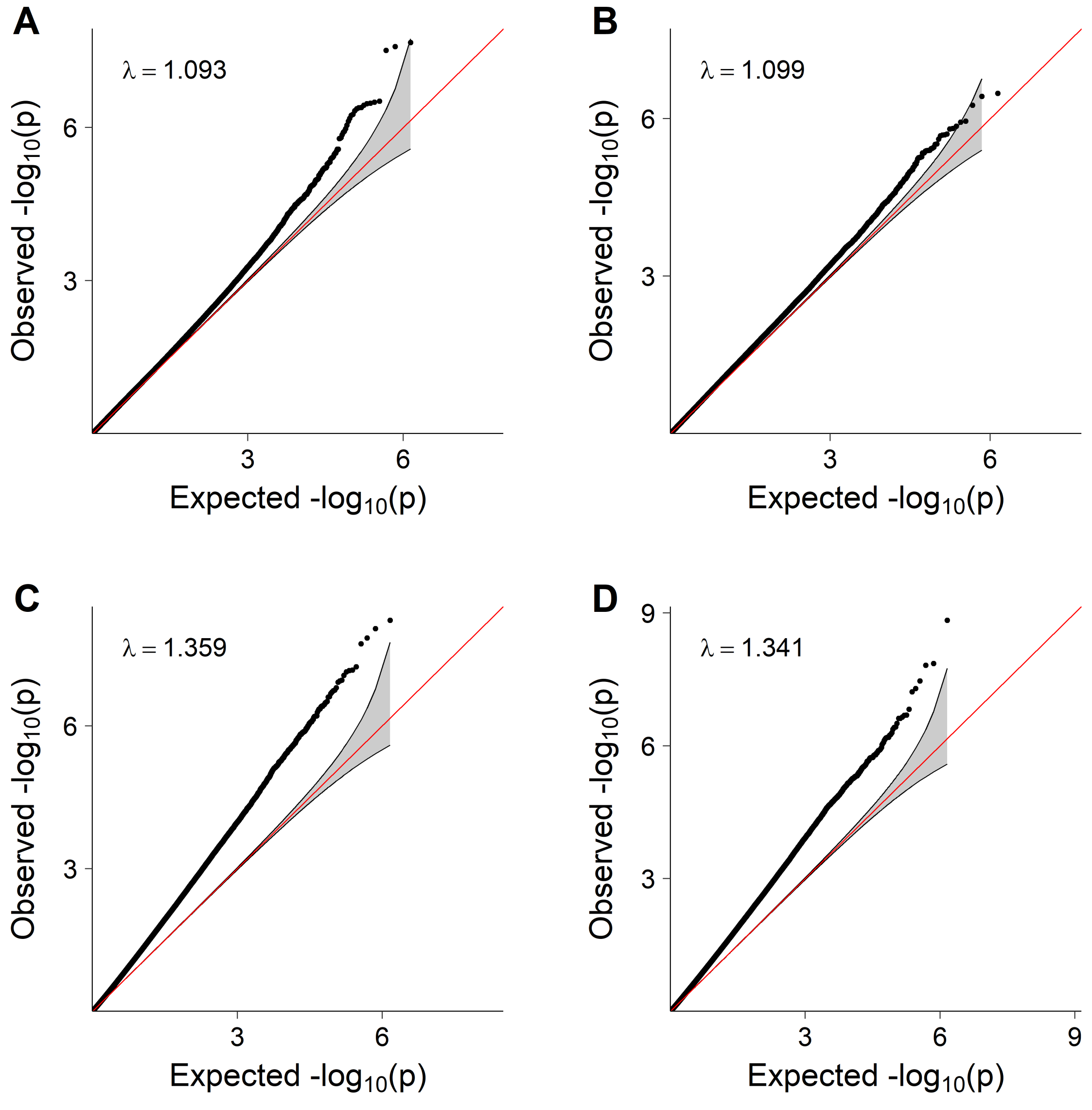

Supplement: S9 Fig — Panels A and C display the parental complementary interaction effects, while panels B and D display the parental threshold-based interaction effects. The shaded area represents a 95% confidence interval band for the null hypothesis p-value distribution. SNPs with MAF lower than 5% are excluded. (TIF) [file pgen.1011952.s012.tif]
