# Supplementary material for: Statistical methods to disentangle genetic effects influencing infertility and early fetal viability with a genome-wide application
Source: PLoS Genet. 2025 Dec 1;21(12):e1011952. doi: 10.1371/journal.pgen.1011952 (PMC12680335; doi:10.1371/journal.pgen.1011952)
Supplement: Attachment — Submitted filename: Response_to_Reviewers.pdf [file pgen.1011952.s016.pdf]

Response to Reviewers

Dear reviewers,

We sincerely appreciate the time you dedicated to reviewing our manuscript. Your thoughtful and constructive feedback has helped us to significantly improve its quality. Below, we provide detailed responses to your comments, including page and line references to the corresponding revisions in the manuscript. Please do not hesitate to contact us with any further questions or suggestions.

Reviewer #1

This manuscript presents a statistical framework to disentangle genetic effects of parents from the fetus on fertilization and early fetal survival. By leveraging data from both ART and non-ART families, the authors identified fetal genetic factors and parental interactions that might be linked to early viability. A key challenge the authors tried to address is the potential aliasing of parental and fetal effects. The statistical model is clearly described with a well-defined set of estimable parameters and assumptions. The authors applied several modeling strategies and compared models with and without parental interaction for the ART and non-ART cohorts. Overall, I think the methodology is well presented. I have a few comments and questions about the interpretation and results that can be improved before considering publication.

Comments

1. It will be helpful to explain more explicitly how the proposed method extends or differs from the Haplin framework for the readers to understand the novelty of the proposed approach.

We have now clarified this at the beginning of the Statistical Models section (page 4, lines 164-168).

2. Can the authors give explicit interpretation of the parental interaction effect estimates? There is some discussion in the Discussion section, but it will be more helpful to have this earlier in the manuscript. What caveats should be kept in mind? Additionally, how do different interaction modeling strategies change the interpretation?

We have added an explicit interpretation of the parental interaction parameter, β_{mf}, in the Statistical Models section (page 6, lines 277-279). We also discuss caveats related to interpreting the parental interaction effect estimates and potential violations of model assumptions (page 6, lines 281-286) and provide further interpretation under different parental interaction modeling strategies (page 6-7, lines 307-317). In the Results section, we now provide interpretation of the parental interaction RR estimates in the non-ART sample (page 8, lines 427-429), and we have added interpretation of the parental interaction RR estimates in the ART sample (page 9, lines 460-462).

3. In Figure 4, modeling both fetal and parental interaction effects results in sometimes same and sometimes opposite directions of relative risk. Does this have biological or statistical implications? More guidance on how to interpret such results would be helpful.

We now provide an explanation of what RR > 1 signifies for the fetal and parental interaction effects in the ART and non-ART samples in the Results section (page 8, lines 409-414). In the Discussion section, we further discuss the interpretation of the directions of relative risk estimates, specifically addressing effects in the same or opposite directions (page 10, lines 520-529).

4. For the identified SNPs, besides which gene regions they are located in or close to, are they located in intronic, exonic, or regulatory regions? What about variant effect type (e.g., missence, nonsence, etc.)? More functional and epigenetic annotations would aid the biological interpretation.

We have added a paragraph describing this in the Discussion section (page 10, lines 509-513). In addition, we have added a column indicating variant type to Table 1.

5. The genomic inflation factors observed in the study are briefly addressed in the Discussion, but more elaboration is needed on the potential causes of inflation or deflation, as well as on the robustness of the findings. Have approaches such as LD score regression been considered to distinguish polygenicity vs. modeling or confounding issues? For the ART cohort, it's likely the sample size is too small for it to have sufficient power, which is a limitation of the study that should be brought up.

We have included comments on potential polygenicity in the Discussion section (page 11, lines 571-574). We also highlight the limitation posed by the relatively small ART sample size (page 12, lines 615-619).

6. Have the authors checked the underlying assumptions in the model with the data?

We have included a thorough evaluation of the four assumptions underlying the model in paragraphs 5-8 in the Discussion section (page 10-11, lines 531-554), discussing their plausibility, whether and how they were tested, and the potential consequences if they are violated.

7. Related to comment 2, in the Discussion section the manuscript talks about interpretations of parental interaction effects and mentions “assuming non-random mating” (line 475), which appears to contradict earlier statements and model assumptions. Clarification is needed regarding the modeling assumptions and interpretation of parameters.

This was a typo; it should read “assuming random mating” and has now been corrected (page 10, line 522). We have also moved the mention of this assumption to the following sentence, as it pertains more to interpretation than to observation. Additionally, we have added further details on modeling assumptions, as well as on parameter interpretation in light of the model assumptions to the Discussion section (see the response to comment 6).

8. Given the multiple interaction modeling approaches (complementary, threshold-based, etc.), in practice, how should one select among them? With many unmeasured confounders and uncontrolled factors, the manuscript would benefit from a clearer discussion of model validity, usability, and interpretability in real use cases.

We have elaborated on model choice in the Discussion section (page 11, lines 579-583). Regarding the overall validity of the parental interaction models, we consider this addressed by the thorough evaluation described in our response to comment 6.

Reviewer #2

Summary

The authors study the question of how to identify whether specific genetic variants influence the probability of fertilization or embryonic survival when parental and fetal genetic effects are analyzed jointly. They develop a statistical model to jointly estimate fetal and parental genetic effects on early fetal viability. The authors then apply this method to a large Norwegian cohort (MoBa), stratifying the data by ART vs. non-ART conception modes, and conduct genome-wide association studies to demonstrate how parental and fetal effects differ between the two groups.

In the non-ART group, the authors identify genome-wide significant fetal effects on fetal survival, primarily for several variants on chromosome 6. These effects remain significant after adjusting for parental interaction effects, supporting their fetal origin. In the ART group, some overlap is observed, and additional associations in male-infertility-related genes (ACTB, FSCN1, RNF216) are reported.

General comment

Estimating the joint effect of parental interaction and fetal genetic effects is a meaningful advancement over traditional triad-based designs and can advance our understanding of the genetic architecture of infertility and early pregnancy loss. This paper is among the first to examine this joint estimation using a family-based study design. The authors' log-linear model builds on the existing framework for case-parent triad analyses available in reference [28] and extends their previous work [24, 27].

The authors identify fetal effects on fetal survival that persist after adjusting for parental interaction effects in the non-ART sample. The evidence is inconclusive regarding whether these effects are present in the ART sample as well.

Nevertheless, the reliability of the observed fetal effects in the non-ART offspring is supported by the sample size, the robustness of the fetal effect estimates due to the case-triad study design, and the relevant genetic context. This may be a major strength of the paper. Analyzing ART and non-ART families separately is a key design choice that—while not causal—enables more interpretable subgroup patterns and avoids potential heterogeneity bias.

My main comment concerns the reproducibility of the results under other statistical models. The statistical model utilized here is natural and relatively simple, but further studies are needed to validate the results, particularly given the model's assumptions (which could be better justified). Some other comments follow.

We have included some notes on the impact of model assumption violations on the fetal effect (minimal) and the parental interaction effect (more substantial) in the Statistical Models section (page 6, lines 281-286). Additionally, we have expanded the Discussion section with paragraphs 5-8 (page 10-11, lines 531-554), each addressing one of the four model assumptions, with particular focus on the parental interaction effect. We suggest potential follow-up analyses, including alternative statistical models in the Discussion section (page 11, lines 591-598 and page 12, lines 628-630).

Other comments

1. On page 6, S denotes the outcome that ` ` the fetus survives pregnancy," which is technically different from the event of "live birth" in Figure 1 (almost very close), as there're cases of death at birth. Please be consistent in terminology including these and "Embryo survival".

We agree that this terminology is somewhat inconsistent. As the reviewer points out, there are a few instances of late stillbirths, as described in the Data section. Therefore, we have reworded the term "live birth" in Figure 1 to "viable birth", to indicate a birth of a fetus that has reached a gestational age compatible with life outside of the uterus. Correspondingly, we have changed terms such as "live birth" or "successful birth" to "viable birth". With this change, we now consider terms such as "fetus survives pregnancy" and "fetal survival" appropriate, as the fetus did indeed survive the pregnancy *up until* the time of birth, regardless of the birth outcome.

We also acknowledge the mixed use of the terms "embryo survival" and "fetal survival". The term "embryo" refers to the earlier part of pregnancy, whereas "fetal" refers to the later. What we are ideally interested in studying is "embryo survival", in order to provide insight into early, undetected pregnancy losses. However, our data do not in fact include *all* surviving embryos, but only those that also survived most of the fetal stage, up until an (almost) viable gestational age. As the majority of pregnancy losses occur early, typically during the embryonic stage, this limitation is likely minimal. Therefore, while "embryo survival" reflects what we set out to investigate, "fetal survival" more accurately describes the analyses possible with the available data, and we have opted to use both terms accordingly. We have introduced this clarification in the Introduction section (page 2, lines 100-103), and we have ensured that the terminology is consistent throughout the text.

2. It seems, from Figure 1, that among the ART parents, there are 4 subgroups coming from Step 1, including the case where both parents are infertile, and the case where both parents are fertile. It seems the knowledge of which subgroup the parents being to is important in the study, but this seems not incorporated in the framework. Am I correct?

Indeed, there are four subgroups, and while they are not fully identifiable within our data, they are to some extent accounted for in the modelling framework. We have added further details on this in the Data section (page 7, lines 357-363).

3. The model relies on assumptions such as Hardy-Weinberg equilibrium, random mating, and equal allele frequencies between sexes (Page 5 lines 1750-177). Please say a few words for each of them to explain why they are (or not) reasonable in the context of the data.

For the fetal effect, which is estimated conditional on the parental genotypes, potential violations of these assumptions are likely to have less impact. This is noted in the Statistical Models section (page 6, lines 282-283), where we also reference our previous work that elaborates on this point. For the parental interaction effect estimation, a thorough evaluation of each of the four assumptions is provided in paragraphs 5-8 in the Discussion section (page 10-11, lines 531-554), as also described in our response to comment 6 from Reviewer 1.

4. Will recent population growth (or change) during the Cohort study (1999-2008) affect any of the model assumption?

We have addressed this point in the Discussion section (page 12, lines 623-626).

5. The ART sample (n ≈ 1,300) is significantly smaller. The manuscript should provide a brief comment on the statistical power to detect interactions in this group, especially given the complex models and low MAFs of many SNPs. Suggestions could include a comment on the minimum detectable effect size in the typical MAF range.

As proof of principle, we performed simulation-based power calculations for the complementary interaction model. Details are provided in the last paragraph of the Results section (page 9, lines 466-476) and illustrated in Supplemental Figure S10, and further commented on in the Discussion section (page 12, lines 615-617).

6. While the MoBa dataset provides a powerful discovery sample, the lack of external replication—either via meta-analysis or independent validation—limits the generalizability of the findings, if possible, discuss potential avenues for follow-up in comparable cohorts.

Please see our response to the Reviewers' main comment.

7. The terms "solid cells" versus "shaded cells" are not totally clear in Figure 2 and the text around it.

We have changed the wording from "shaded cells" to "patterned cells (diagonal striping)" in the Figure 2 caption, and to "patterned cells" in the text referring to Figure 2.

8. On Page 9 line 368, [htpb] maybe deleted.

This has been deleted (page 8, line 420); thank you for pointing it out.

9. Citation to the paper of Wilcox et al is missing on Page 13 line 450.

Due to adjustments in the Discussion section, the citation to Wilcox *et al.* (PMID: 9801020) now appears as reference [56] (page 10, line 548).

10. The figures are generally clear, but a composite figure directly comparing the most significant loci in ART vs non-ART may enhance interpretation.

We have included two Miami plots comparing the parental interaction effect results between the ART and non-ART samples as a new Supplemental Figure S8, which is referenced in the Results section (page 9, lines 443-444).

Reviewer #3

Thank you for the opportunity to review this manuscript. The authors analyzed the ART and non-ART sample and compared the results between two groups, between with and without adjustment for interaction effects, and among different choices of interaction effects. Overall, the manuscript is well written and provides valuable insights. However, some issues need to be addressed before publication.

1. The model structure involves P(A|M,F,C), while the model is fitted separately for A=0 and A=1. As the interaction effects are used to estimate P(A|M,F,C), I am not confident in the reliability of the coefficient estimate for the interaction effects, when fitting separately for A=0 and A=1. In other words, since the model indicates that beta_mf is the coefficient for the effect of the interaction term on the risk of ART, it is unreasonable to estimate the coefficient only using the ART or non-ART samples.

As you point out, there are challenges to estimating the MxF interaction based only on the ART samples. It relies to a larger extent on the assumptions we make. In particular, the assumption of random mating between parents. Given this, though, we do believe that one obtains valid estimates from the ART triads alone, since the beta_mf coefficient is identifiable from the distribution of M,F conditional on A. The basis for this is the same argument as in "case-only" analyses, where one can estimate a GxE interaction based only on cases, under the assumption that G and E are independent in the population. To make this clearer to the reader, we have now re-written and slightly expanded the part of the Statistical Models section developing that part of the models (page 5, lines 222-223 and lines 225-230), also being more explicit in our reference to the papers by Andrieu and Goldstein (1998) and Piegorsch, Weinberg, Taylor (1994) where the similar GxE case-only design is described (page 5, lines 231-235). We hope this has improved the presentation.

2. In all the formulas derived in Section 4.1, authors used the multiplicative interaction effect while in real data analysis, they instead used complementary and threshold-based parental interaction effects. It is suggested to use complementary and threshold-based parental interaction effects in the formulas derived in Section 4.1 or include more details, such as the formulas for these two interaction effects.

We have now added specific details on how to code the dominant interaction effect, as an illustration, in the Statistical Models section (page 6, lines 307-311). The complementary and threshold-based models then follow the same strategy as described (page 7, lines 315-318), but we have left out the detailed formulation since it is clearly described in our previous (referenced) paper and we feel that the methods description in the current paper is already quite long and complex.

3. On page 5, Paragraph 2, the notations are not clear to me. It seems that A_h denotes different alleles (rather than a variable), but A_i, A_j, ..., appears to be variables.

A_h, A_i, and A_j all denote alleles, with h, i, and j in {1,2,...,H}. We have written the corresponding genotypes not as A_i A_j but rather as (A_i,A_j) to specify that they are ordered. With M=(A_i,A_j) we have used the (somewhat simplified) notation that P(M) denotes the probability that a given mother has the ordered genotype M. We agree that this "shortcut" notation might make the A's look a bit like events, not alleles, so we have now re-written this paragraph, hopefully making this and some other aspects clearer (page 4, lines 172-182).

4. Considering there are only 842 samples in the ART sample, do you worry about the reliability of your analysis in the ART sample? In addition, the significant discrepancy between the sample sizes in the two groups may affect the comparison between results from the ART and non-ART sample. More discussion on it is needed.

We have highlighted some limitations due to the small ART sample size in the Results section (page 9, lines 466-476), and further commented on this, as well as the reliability of comparisons between the ART and non-ART samples, in the Discussion section (page 12, lines 615-619).

5. Do you have any explanation for the lack of overlap between the SNPs selected by the complementary and threshold-based parental interaction effects? For example, if the threshold relaxed, is there any overlap?

We have commented on the observation that the complementary and threshold models show overlap in the ART sample but not in the non-ART sample in the Discussion section (page 11, lines 608-612). In addition, we have discussed the theoretical overlap between the complementary and threshold model under alternative threshold values (page 11, lines 588-591).

6. Before Table 1, “[htbp]” should be removed.

This has been removed (page 8, line 420), thank you for pointing it out.
