# Supplementary material for: Statistical methods to disentangle genetic effects influencing infertility and early fetal viability with a genome-wide application
Source: PLoS Genet. 2025 Dec 1;21(12):e1011952. doi: 10.1371/journal.pgen.1011952 (PMC12680335; doi:10.1371/journal.pgen.1011952)
Supplement: Attachment — Submitted filename: Referee report submit.docx [file pgen.1011952.s015.docx]

**Referee Report for PLOS Genetics**
**Manuscript Title:** *Statistical methods to disentangle genetic effects influencing infertility and early fetal viability with a genome-wide application*

**Summary**

The authors study the question of how to identify whether specific genetic variants influence the probability of fertilization or embryonic survival when parental and fetal genetic effects are analyzed jointly. They develop a statistical model to jointly estimate fetal and parental genetic effects on early fetal viability. The authors then apply this method to a large Norwegian cohort (MoBa), stratifying the data by ART vs. non-ART conception modes, and conduct genome-wide association studies to demonstrate how parental and fetal effects differ between the two groups.

In the non-ART group, the authors identify genome-wide significant fetal effects on fetal survival, primarily for several variants on chromosome 6. These effects remain significant after adjusting for parental interaction effects, supporting their fetal origin. In the ART group, some overlap is observed, and additional associations in male-infertility-related genes (*ACTB*, *FSCN1*, *RNF216*) are reported.

**General comment**

Estimating the joint effect of parental interaction and fetal genetic effects is a meaningful advancement over traditional triad-based designs and can advance our understanding of the genetic architecture of infertility and early pregnancy loss. This paper is among the first to examine this joint estimation using a family-based study design. The authors’ log-linear model builds on the existing framework for case-parent triad analyses available in reference [28] and extends their previous work [24, 27].

The authors identify fetal effects on fetal survival that persist after adjusting for parental interaction effects in the non-ART sample. The evidence is inconclusive regarding whether these effects are present in the ART sample as well.

Nevertheless, the reliability of the observed fetal effects in the non-ART offspring is supported by the sample size, the robustness of the fetal effect estimates due to the case-triad study design, and the relevant genetic context. This may be a major strength of the paper. Analyzing ART and non-ART families separately is a key design choice that—while not causal—enables more interpretable subgroup patterns and avoids potential heterogeneity bias.

My main comment concerns the **reproducibility** of the results under other statistical models. The statistical model utilized here is natural and relatively simple, but further studies are needed to validate the results, particularly given the model’s assumptions (which could be better justified). Some other comments follow.

**Other comments**

1. On page 6, S denotes the outcome that ``the fetus survives pregnancy,” which is technically different from the event of “live birth” in Figure 1 (almost very close), as there’re cases of death at birth. Please be consistent in terminology including these and “Embryo survival”.
2. It seems, from Figure 1, that among the ART parents, there are 4 subgroups coming from Step 1, including the case where both parents are infertile, and the case where both parents are fertile. It seems the knowledge of which subgroup the parents being to is important in the study, but this seems not incorporated in the framework. Am I correct?
3. The model relies on assumptions such as Hardy-Weinberg equilibrium, random mating, and equal allele frequencies between sexes (Page 5 lines 1750-177). Please say a few words for each of them to explain why they are (or not) reasonable in the context of the data.
4. Will recent population growth (or change) during the Cohort study (1999-2008) affect any of the model assumption?
5. The ART sample (n ≈ 1,300) is significantly smaller. The manuscript should provide a brief comment on the statistical power to detect interactions in this group, especially given the complex models and low MAFs of many SNPs. Suggestions could include a comment on the minimum detectable effect size in the typical MAF range.
6. While the MoBa dataset provides a powerful discovery sample, the lack of external replication—either via meta-analysis or independent validation—limits the generalizability of the findings, if possible, discuss potential avenues for follow-up in comparable cohorts.
7. The terms “solid cells” versus “shaded cells” are not totally clear in Figure 2 and the text around it.
8. On Page 9 line 368, [htpb] maybe deleted.
9. Citation to the paper of Wilcox et al is missing on Page 13 line 450.
10. The figures are generally clear, but a composite figure directly comparing the most significant loci in ART vs non-ART may enhance interpretation.
